# Monitoring of Hepatitis E Virus in Wild Lagomorphs in Spanish Mediterranean Ecosystems

**DOI:** 10.1155/2023/7947220

**Published:** 2023-04-19

**Authors:** Sabrina Castro-Scholten, Javier Caballero-Gómez, Antonio Rivero-Juarez, David Cano-Terriza, Félix Gómez-Guillamón, Débora Jiménez-Martín, Antonio Rivero, Ignacio García-Bocanegra

**Affiliations:** ^1^Departamento de Sanidad Animal, Grupo de Investigación en Sanidad Animal y Zoonosis (GISAZ), UIC Zoonosis y Enfermedades Emergentes ENZOEM, Universidad de Córdoba, 14004 Córdoba, Spain; ^2^Grupo de Virología Clínica y Zoonosis, Unidad de Enfermedades Infecciosas, Instituto Maimónides de Investigación Biomédica de Córdoba (IMIBIC), Hospital Universitario Reina Sofía, Universidad de Córdoba, Córdoba 14014, Spain; ^3^CIBERINFEC, ISCIII CIBER de Enfermedades Infecciosas, Instituto de Salud Carlos III, Madrid 28029, Spain; ^4^Programa de Vigilancia Epidemiológica de la Fauna Silvestre (PVE), Consejería de Sostenibilidad, Medio Ambiente y Economía Azul, Junta de Andalucía, Málaga 29002, Spain

## Abstract

Hepatitis E virus (HEV) is an emerging zoonotic pathogen in Europe. Even though swine species are considered the main host of the zoonotic HEV-3 genotype, rabbits are recognized as the main reservoir of the divergent HEV-3ra subtype. However, the role of wild lagomorphs in the epidemiology of this virus in Mediterranean ecosystems is under debate. The aims of this study were to assess exposure of HEV in wild rabbit (*Oryctolagus cuniculus*) and Iberian hare (*Lepus granatensis*) populations in southern Spain and to determine potential risk factors associated with HEV exposure in these species. Between 2018 and 2021, blood and fecal samples from 370 wild rabbits and 60 Iberian hares were collected. A total of 29 (6.7%; 95%CI: 4.4–9.1) out of 430 sampled animals showed anti-HEV antibodies. By species, the seroprevalences in wild rabbit and Iberian hare were 6.8% (29/370; 95%CI: 4.2–9.3) and 6.7% (4/60; 95%CI: 0.4–13.0), respectively. Seropositive animals were detected on 17 (26.2%; 95%CI: 15.4–36.8) of the 65 sampled hunting estates. The generalized estimating equations model showed that geographical area was a risk factor potentially associated with HEV exposure in wild lagomorphs in the study region. HEV RNA was not detected in any of the 242 (0.0%; 95%CI: 0.0–1.5) fecal samples tested. This is the first large-scale serosurvey performed in wild rabbits in the Iberian Peninsula and in Iberian hares worldwide. Our results provide evidence of low, widespread, and heterogeneous distribution of HEV among wild rabbit and Iberian hare populations in Spanish Mediterranean ecosystems, which indicates a limited role of wild lagomorphs in the maintenance of the virus and a low risk of transmission of HEV to other species, including humans.

## 1. Introduction

*Paslahepevirus balayani* (also known as hepatitis E virus (HEV); family *Hepeviridae*) is the main cause of human acute viral hepatitis worldwide [1]. Eight different genotypes of HEV have been recognized so far (HEV-1 to 8), of which HEV-3, HEV-4 and HEV-7 are zoonotic. Among them, HEV-3 is the genotype with the greatest geographical distribution, being the most prevalent in many high-income regions, including Europe [2]. In this continent, HEV-3 human cases have considerably increased during the last few years, and contact with infected animals and the consumption of raw or undercooked animal products have been shown to be the major transmission routes of HEV infection [3]. Although wild boar (*Sus scrofa*) and domestic pig (*Sus scrofa domesticus*) are recognized as the main hosts of HEV-3 [4], rabbits, which are also susceptible to swine HEV-3 related strains [5, 6], represent the major reservoir of the divergent but also emergent and zoonotic HEV-3ra subtype [7].

Since the first description of HEV-3ra in domestic and wild rabbits in France in 2012 [8], HEV RNA and anti-HEV antibodies have been detected in lagomorphs in several European countries, including France [9], United Kingdom, Italy [6, 10], The Netherlands [11], Poland [12], and Germany [5, 13–15] with prevalence and seroprevalence values ranged from 5.0% to 60.0% and from 2.6% to 37.3%, respectively. In the Iberian Peninsula, the European wild rabbit (*Oryctolagus cuniculus*) and the Iberian hare (*Lepus granatensis*) are two endemic species, being the most important small game lagomorph species in terms of abundance and hunting interest. These species are considered an important source of food for humans, especially in rural areas and usually for self-consumption, as rabbit meat is one of the most nutritional white meats [16]. Over the last decade, circulation of HEV has been confirmed in sympatric wildlife from this European region, such as wild boar (*Sus scrofa*) [17], red deer (*Cervus elaphus*) [18], and also the Iberian lynx (*Lynx pardinus*) [19], whose staple prey is the European wild rabbit. Even though active HEV infection was not found in wild lagomorphs in southern Spain [20], only a single local study has assessed HEV exposure in a limited number of wild lagomorphs in the Iberian Peninsula (southern Portugal), detecting seropositivity [21]. Therefore, the involvement of these species in the epidemiology of HEV in the Iberian Peninsula is under debate. The aims of the present study were to assess the role of wild lagomorphs in the epidemiology of HEV in Mediterranean ecosystems of southern Spain and to determine potential risk factors associated with HEV exposure in these species.

## 2. Material and Methods

### 2.1. Study Design and Sampling

A cross-sectional study was carried out in Andalusia (southern Spain; 36°N-38°60′N, 1°75′W-7°25′W), one of the most important regions in Spain in terms of small game population [22]. About 1.4 million wild rabbits and 251 thousand hares are harvested annually in Andalusia, being the second Spanish community with the highest number of lagomorphs hunted annually [22, 23]. The Andalusian Mediterranean ecosystems are characterized by the “dehesa” agroforestry system interspersed with Mediterranean forest, where different land uses such as agriculture, farming, and/or hunting are simultaneously exploited. The climate is a continental thermos Mediterranean climate with hot, dry summers and mild winters. The western region presents higher mean humidity and less extreme mean temperatures than the central and eastern regions.

**Figure 1:**
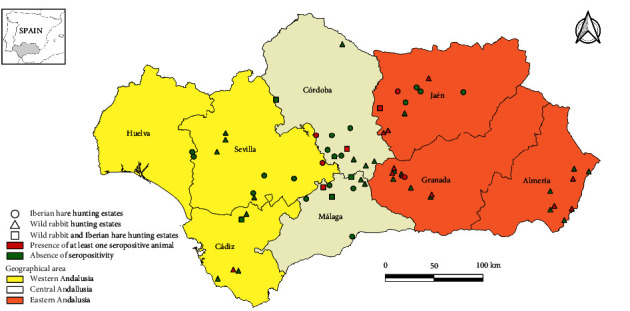
Map of the study area (Andalusia, southern Spain) showing the location of the sampled wild lagomorphs hunting estates.

Whenever possible, 60 wild rabbits were sampled per province in order to ensure a 95% probability of detecting at least one positive animal, assuming a minimum seroprevalence of 5% in each province [24]. In addition, for each province, sampling sites (hunting states) were randomly selected, and between 1 and 15 (mean: 8.6), wild rabbits were kindly provided by hunters in each of the hunting estates to collect samples (Figure 1). Between 2020 and 2021, serum and fecal samples were collected from a total of 370 wild rabbits sampled in 43 hunting estates. In addition, serum and feces from 60 Iberian hares were taken between 2018 and 2021 in 28 hunting estates in the same study region using a convenience sampling.

Blood samples from all animals were taken from the heart or thoracic cavity, and sera were then obtained after blood centrifugation at 400*g* for 10 min. Feces were collected directly from the rectum. Samples were stored at −80°C until laboratorial analysis.

Information about each animal, including location, sampling date, age, and sex, was recorded whenever possible (Table 1). Bodyweight and length were used as indicators of age [25]. Three groups of age were considered: young (<40 days old), subadult (from 40 days to 8 months), or adult (over 8 months). Epidemiological data related to hunting estates were also gathered through personal interviews with the gamekeepers using a standardized questionnaire (Table 1).

### 2.2. Laboratory Analysis

The presence of anti-HEV antibodies was assessed using a commercial double-antigen multispecies ELISA (HEV ELISA 4.0v; MP Diagnostics, Illkirch, France), in accordance with the manufacturer's instructions. Samples were considered positive when the optical density (OD) at 450 nm of the sample was superior to the cut-off value (cut-off value = mean OD of the negative control + 0.2). This assay is based on the highly conserved recombinant protein ET2.1 [26] and detects the presence of total antibodies (IgM, IgG, and IgA) against the virus in sera or plasma in a wide range of animal species.

RNA from feces of wild lagomorphs from seropositive hunting states was extracted using the IndiSpin Pathogen Kit (Indical Biosciences, Germany) following the manufacturer's instructions. For HEV RNA detection, two RT-PCR assays were carried out in parallel. A broad-spectrum real-time RT-PCR (CFX connect real-time PCR system) capable of detecting all *P. balayani*, genotypes, including HEV-3ra subtype, with the QIAGEN one-step RT-PCR kit (QIAGEN, Hilden, Germany) was used as previously described [27]. In addition, a nested broad-spectrum RT-PCR that is capable to amplify the four genera of hepevirus was also carried out according with Johne et al. [28]. For the first round, the QIAGEN one-step RT-PCR kit was used whereas the nested PCR was carried out with the premixed 2X solution Taq DNA polymerase, dNTPs, and reaction buffer kit (Promega). The second PCR products were examined on 1.5% agarose gel stained with RedSafe™ Nucleic Acid Staining Solution.

### 2.3. Statistical Analyses

The prevalence and seroprevalence against HEV was determined from the proportion of positive and seropositive animals, respectively, to the total number of examined, using the two-sided exact binomial test, 95% CI. Firstly, associations between results and independent variables were screened using the Pearson's Chi-square or Fisher's exact test, as appropriate. All variables with a *p* < 0.05 in the bivariate analysis were selected for further analyses. Collinearity between pairs of variables was tested by Cramer's V coefficient. Finally, a generalized estimating equation (GEE) analysis was carried out to study the effect of the variables selected based on the bivariate analysis. The number of positive animals was assumed to follow a binomial distribution, and “hunting estate” was included as the subject variable. The model was rerun until all remaining variables showed statistically significant values (*p* < 0.05). SPSS 25.0 software (Statistical Package for Social Sciences, Inc., Chicago, IL, USA) was used for all statistical analyses.

## 3. Results and Discussion

In recent decade, new viral strains of HEV have emerged in different European countries, which may have important clinical and epidemiological implications [29–32]. Since 2012, an increasing number of HEV-3ra human cases has been confirmed in France, Belgium, Switzerland, and, recently, also in Spain [8, 29, 33–35], and the high homology between HEV-3ra isolates from humans and rabbits supports the hypothesis of zoonotic transmission linked to consumption of these animals [3]. In addition, HEV-3ra seems to produce chronic hepatitis E cases in immunosuppressed patients more often than other subtypes [33]. In this context, understanding the role of lagomorphs in the epidemiology of HEV-3ra is an important key issue to control this emerging genotype.

The present study provides new epidemiological data on HEV in wild lagomorph populations in Mediterranean ecosystems of southern Spain. The major strengths of the survey include a large sample size, which is representative of the whole study area. A total of 29 (6.7%; 95%CI: 4.4–9.1) out of 430 sampled animals showed anti-HEV antibodies, which confirm that wild lagomorphs are naturally exposed to HEV in Iberian Mediterranean ecosystems. The distribution of seropositivity according to the explanatory variables is shown in Table 1. The seroprevalence in wild rabbit was 6.8% (29/370; 95% CI: 4.2–9.3). Our result is consistent with that previously found in this species in Australia (9.1%; 33/362) [36] and slightly higher to those reported in the United Kingdom (3.3%; 1/30) [6] and also in a local study conducted in southern Portugal, where 3 out of 74 showed anti-HEV antibodies (4.1%) [21]. By contrast, higher seropositivity values were obtained in wild rabbits in Japan (33.3%; 20/60) [37], Germany (30.8%; 4/13–37.3%; 47/126) [5, 13], Italy (42.9%; 15/35) [6], and Burkina Faso (60.0%; 60/100) [38].

In the present study, seropositivity was detected in four (6.7%; 95%CI: 0.4–13.0) of the 60 Iberian hares sampled. It should be noted that only a very few numbers of serosurveys have been conducted to date in hares worldwide. Two of them were carried out in Germany in European brown hare (*Lepus europaeus*) and found lower seroprevalences (2.2%; 14/624−2.6%; 25/944) [5, 15], whereas higher seropositivity values were found in *Lepus africana* in Burkina Faso (52.6%; 10/19) [38]. Comparisons between studies should be made with caution, given the differences in the serological methods used, study design and species, and number of animals sampled. Nevertheless, we would like to state that the seroprevalence of HEV in wild rabbits and Iberian hares in the study area should be considered low.

Statistical differences among ages were not found (*p*=0.228) (Table 1). In line, seropositivity was detected in 6.0% (7/117) of subadults and 12.8% (5/39) of young lagomorphs in all the years of the study period. Although the presence of maternal antibodies in yearling individuals cannot be ruled out, our results suggest endemic HEV circulation in wild lagomorph populations in Iberian Mediterranean ecosystems during the study period. Seropositive animals were found in all the provinces of the study area and in 17 (26.2%; 95%CI: 15.4–36.8) of the 65 sampled hunting estates (Figure 1).

Although variables related with the hunting estate (“geographical area,” “presence of wildcat,” “presence of goat,” “presence of swamps,” and “lagomorph density”) showed association (*p* < 0.05) with the dependent variable in the bivariate analyses (Table 1), only the variable “geographical area” was retained in the GEE model. Significantly higher seropositivity was detected in eastern (11.2%; 95%CI: 6.9–15.5; *p*=0.009; OR = 6.5 [1.6–26.4]) compared to central Andalusia (2.2%; 95%CI: 0.0–4.7) but not with western region (3.4%; 95%CI: 0.0–7.1; *p*=0.456; OR = 1.9 [0.3–10.1]). These findings denote a widespread but not homogeneous distribution of HEV in wild rabbit and Iberian hare populations from southern Spain.

Experimental studies reported that HEV can be detected in lagomorphs' feces between two- and ten-weeks postinfection [39]. Previous studies have confirmed natural circulation of HEV in wild lagomorphs with prevalence values that ranged between 1.7% and 16.0% [8, 11, 37]. In the present study, none of the 242 (0.0%; 95%CI: 0.0–1.5) feces analyzed were positive to HEV RNA, which indicate a limited active HEV circulation in wild lagomorphs in southern Spain. Our results are also consistent with the absence of infection reported previously in liver from these species in the same study region [20] and confirm a limited risk of transmission of HEV from wild lagomorphs to other sympatric species, including humans.

In conclusion, the serological results obtained in the present study indicate natural exposure to HEV but low, widespread, and heterogeneous viral circulation in wild rabbits and Iberian hare populations in southern Spain. The low seroprevalence as well as the absence of active HEV infection point that these wild lagomorphs play a limited role in the epidemiology of this virus in Mediterranean ecosystems of southern Spain. Further studies in different regions of the Iberian Peninsula are warranted to get a deeper and broader understanding of the epidemiological situation of HEV in these lagomorph species.

## Figures and Tables

**Table 1:** Distribution of the seroprevalence against HEV in wild lagomorphs in Andalusia (southern Spain) by animal and hunting estate categories and results of the bivariate analysis.

Variables	Categories	No. positives/overall^*∗*^	Seroprevalence (%)	*P*
Species	Wild rabbit	25/370	6.8	0.620
	Iberian hare	4/60	6.7	

Age	Adult	15/265	5.7	0.228
	Subadult	7/117	6.0	
	Young	5/39	12.8	

Sex	Male	14/213	6.6	0.536
	Female	13/206	6.3	

Kidney fat index	0	2/56	3.6	0.371
	1	9/122	7.4	
	2	7/98	7.1	
	3	8/67	11.9	

Bodyweight (kg)	0.1–0.9	12/121	9.9	0.274
	1.0–1.1	7/141	5.0	
	1.2–3.1	6/97	6.2	

Body length (cm)	28–37	12/142	8.5	0.836
	38–40	8/121	6.6	
	41–56	6/86	7.0	

Geographical area	Western	3/89	3.4	0.002
	Central	3/136	2.2	
	Eastern	23/205	11.2	

Hunting season	2017/2018	0/1	0.0	0.925
	2018/2019	1/10	10.0	
	2019/2020	2/24	8.3	
	2020/2021	25/365	6.8	
	2021/2022	1/30	3.3	

Burrow density	High	22/348	6.3	0.615
	Medium	0/12	0.0	
	Low	3/38	7.9	

Presence of ticks	Yes	20/293	6.8	0.313
	No	5/105	4.8	

Presence of fleas	Yes	21/274	7.7	0.066
	No	4/124	3.2	
Last restocking (months)	No	12/244	4.9	0.277
	≤6	2/39	5.1	
	6–12	5/65	7.7	
	≥12	6/50	12.0	

Origin of the restocking	Same game estate	11/114	9.6	0.425
	Another game estate	2/32	6.3	

Cases of myxomatosis in the last year	Yes	24/356	6.7	0.233
	No	1/42	2.4	

Cases of RHD in the last year	Yes	19/304	6.3	0.563
	No	6/94	6.4	

Fenced hunting estate	Yes	0/26	0.0	0.175
	No	25/372	6.7	

Distance to urban areas (Km)	<10	25/352	7.1	0.105
	10–20	0/32	0.0	

Presence of wild boar (*Sus scrofa*)	Yes	17/143	10.6	0.261
	No	6/78	7.1	

Presence of red deer (*Cervus elaphus*)	Yes	0/35	0.0	0.071
	No	29/351	7.6	

Presence of wildcat (*Felis* silvestris)	Yes	14/135	10.4	0.016
	No	11/263	4.2	

Presence of Iberian lynx (*Lynx pardinus*)	Yes	3/24	12.5	0.184
	No	22/374	5.9	

Presence of badger (*Meles meles*)	Yes	22/348	6.3	0.614
	No	3/50	6.0	

Presence of marten (*Martes foina*)	Yes	19/256	7.4	0.148
	No	6/142	4.2	

Presence of weasel (*Mustela nivalis*)	Yes	19/301	6.3	0.591
	No	6/97	6.2	

Presence of polecat (*Mustela putorius*)	Yes	11/198	5.6	0.350
	No	14/200	7.0	

Presence of domestic cat (*Felis silvestris catus*)	Yes	24/368	6.5	0.420
	No	1/30	3.3	

Presence of dog (*Canis familiaris*)	Yes	22/308	7.1	0.142
	No	3/90	3.3	

Presence of cattle (*Bos taurus*)	Yes	0/33	0.0	0.097
	No	24/335	7.2	

Presence of goat (*Capra aegagrus hircus*)	Yes	15/152	9.9	0.025
	No	9/216	4.2	

Presence of sheep (*Ovis aries*)	Yes	13/199	6.5	0.582
	No	11/169	6.5	

Presence of farmed rabbit (*Oryctolagus cuniculus*)	Yes	2/14	14.3	0.217
	No	23/384	6.0	

Presence of domestic pig (*Sus scrofa domesticus*)	Yes	0/26	0.0	0.162
	No	24/343	7.0	

Presence of rabbit feeders	Yes	14/214	6.5	0.492
	No	11/184	6.0	

Feed supplementation in rabbits	Yes	5/144	3.5	0.060
	No	20/254	7.9	

Presence of swamps	Yes	7/49	14.3	0.023
	No	18/349	5.2	

Presence of waterholes	Yes	14/174	8.0	0.142
	No	11/224	4.9	

Presence of sources	Yes	10/340	7.5	0.313
	No	15/264	5.7	

Presence of troughs	Yes	18/323	5.6	0.093
	No	7/63	11.1	

Presence of streams	Yes	13/187	7.0	0.377
	No	12/211	5.7	

The hunting estate is weeded	Yes	8/71	11.3	0.057
	No	17/327	5.2	

Cleaning watering places	Yes	9/126	7.1	0.389
	No	16/272	5.9	

Presence of artificial burrows	Yes	3/33	9.1	0.344
	No	22/365	6.0	

Crops intended for hunting	Yes	11/161	6.8	0.431
	No	14/237	5.9	

Lagomorph density^*∗∗*^ (animal hunted/km2)	High (51–100)	5/25	20.0	0.037
	Medium (26–50)	5/36	13.9	
	Low (11–25)	2/38	5.3	
	Very low (0–10)	4/94	4.3	

^*∗*^Missing values omitted. ^*∗∗*^Calculated from the average number of animals hunted in the last 10 years in the sampled hunting estate.

## Data Availability

The data that support the findings of this study are available from the authors upon reasonable request.
